# Prediction of the Recurrence of Non-Functioning Pituitary Adenomas Using Preoperative Supra-Intra Sellar Volume and Tumor-Carotid Distance

**DOI:** 10.3389/fendo.2021.748997

**Published:** 2021-09-30

**Authors:** Wenli Chen, Mengqi Wang, Chengbin Duan, Shun Yao, Haosen Jiao, Zongming Wang, Bin Hu, Zhigang Mao, Yonghong Zhu, Haijun Wang

**Affiliations:** ^1^ Center for Pituitary Surgery, Department of Neurosurgery, The First Affiliated Hospital, Sun Yat-sen University, Guangzhou, China; ^2^ Department of Histology and Embryology, Zhongshan School of Medicine, Sun Yat-Sen University, Guangzhou, China

**Keywords:** non-functioning pituitary adenomas, V-D value, recurrence, prediction, pre-operative

## Abstract

**Background:**

Currently, it is difficult to estimate the possibility of recurrence of nonfunctioning pituitary adenomas (NFPAs). Markers such as Ki-67 or transcription factors rely on postoperative pathology, while few indices can be used for preoperative prediction. Therefore, we aimed to investigate the predictive effectiveness of supra-intrasellar volume and tumor-carotid distance based on measurements derived from preoperative magnetic resonance imaging (MRI) data.

**Method:**

Ninety-eight cases of NFPAs were evaluated, along with their clinical characteristics and MRI features. Four radiologic indices were analyzed, including intrasellar tumor volume, suprasellar tumor volume, maximum horizontal tumor diameter, and intercarotid distance. The ratio of supra-intrasellar volume and ratio of tumor-carotid distance were measured using 3D Slicer software, and the sum of two ratios was defined as the V-D value. The correlation between recurrence and multiple factors was analyzed using univariate and multivariate logistic regression and Kaplan-Meier analysis, and ROC curves were used to estimate the prognostic performance of radiologic measurements in NFPAs.

**Result:**

The supra-intrasellar volume ratio, tumor-carotid distance ratio and V-D value were significantly correlated with the recurrence of NFPAs. The predictive importance of the V-D value reached 84.5%, with a sensitivity of 83.7% and specificity of 67.3%. The cutoff limit of the V-D value was 1.53, and patients with V-D values higher than 1.53 tended to relapse much earlier.

**Conclusion:**

The V-D value has predictive importance for the recurrence of NFPAs preoperatively. Patients with higher V-D values will undergo recurrence earlier and should be given greater consideration in terms of surgery and follow-up time.

## Introduction

Nonfunctioning pituitary adenomas (NFPAs), accounting for 15% - 54% of all types of pituitary adenomas (PAs) (1), are characterized by a lack of hormonal oversecretion except for the high level of prolactin, as well as subclinical or silent hormone dysfunction caused by the “pituitary stalk effect” (2). Over 60% of NFPAs are macroadenomas (diameter over 10 mm), and 57% are invasive (3). Most patients present with headaches, hormone disorders, visual impairment, or cranial nerve palsy. The diagnosis of most NFPAs is usually delayed by a mean time of 1.96 ± 2.9 years due to the absence of endocrine symptoms (4, 5). Complete surgical resection can only be achieved in approximately 40–50% of all patients, although surgery is still the first-line strategy in NFPAs (6). Over 10–20% of cases with gross tumor resection will experience relapse 5–10 years postoperatively. This number rises to 40% and 50% in 5 years and 10 years, respectively, if there is a residual tumor after operation (7).

However, there is a great degree of heterogeneity among cases in terms of tumor recurrence. According to the 2017 WHO classification of brain tumors, most NFPAs are considered gonadotropin adenomas with a low chance of relapse. However, some subtypes of NFPAs, such as silent ACTH adenoma, Pit-1-positive adenomas or plurihormonal adenomas, have a high risk of recurrence but can only be affirmed by postoperative pathological tests. The monoclonal antibody Ki-67 is often used as a tool to aid in recurrence prediction, but its accuracy is still controversial (1). Currently, there are few reliable indices for the prediction of the recurrence of NFPAs (8). The preoperative evaluation still relies on classic methods, including the Hardy-Wilson, and Knosp classifications. Some researchers have reported that these two systems are not effective for precise prediction of tumor recurrence (9–11). The Hardy–Wilson system mainly reflects the longitudinal extension of the tumor, while the Knosp system reflects horizontal invasiveness.

Invasion into the cavernous and/or sphenoid sinus has been proven to be effective in the prediction of recurrence in NFPAs (12) but quantitative analysis is necessary for precise evaluation. Yan et al., have already used the intercarotid distance for the prediction of total resection of PAs, which is closely related to recurrence (13). Therefore, we aim to develop a brief and effective evaluation system using intercarotid distance and supra-intrasellar volume for quantifying the horizontal and longitudinal extension of the tumor. Although many predictive models have already been built, our study aims to provide an evaluation system that is easy to manipulate and use routinely.

## Materials and Methods

### Patient Samples

A total of 206 patients diagnosed with NFPAs by preoperative pituitary hormones, clinical behavior and postoperative immunohistochemistry were included in this study. After removal of the cases without adequate clinical or radiologic data, 98 cases who had undergone endoscopic trans-sphenoidal surgery in the First Affiliated Hospital, Sun Yat-sen University (Guangzhou, China) between January 2012 and December 2019. were finally selected. According to the tumor size, NFPAs can be classified into small adenomas (tumor largest diameter < 1.5 cm), large adenomas (1.5 <= tumor largest diameter < 3 cm) and giant adenomas (≥ 3 cm). Knosp and Hardy–Wilson grades were also evaluated by 2 doctors of the neurosurgical department independently.

Hormone-secreting PAs, NFPAs combined with other tumors and recurrent NFPAs were excluded from this study. The laboratory examination of serum hormones including prolactin (PRL), growth hormone (GH), insulin-like growth factor-1 (IGF-1), thyroid-stimulating hormone (TSH), luteinizing hormone (LH), and follicle-stimulating hormone (FSH) was performed on the first day of attendance. All kinds of functioning pituitary adenomas were excluded according to the hormone levels and pathologic results. To prevent the potential errors caused by the “hook effect”, serial dilution of the samples and re-measuring was performed.

Tumor recurrence was defined as (1): a tumor larger than 0.1 cm^3^ after total resection or (2) a residual tumor increased more than 25% after subtotal resection, during follow-up. Recurrence time was defined as the duration from the time of the first surgery to the first time of positive radiological findings. This study was approved by the Ethics Committee of The First Affiliated Hospital of Sun Yat-Sen University and was done in accordance with the Declaration of Helsinki.

### Imaging Protocol

All patients had undergone sellar MRI using a high-field magnetic system (3.0T, *Magnetom Verio*, Siemens Healthcare, Erlangen, Germany) with a 64-channel head/neck coil. The regular sequences included coronal and sagittal T1-weighted turbo spin-echo (TSE) with and without contrast (TR =550 ms, TE = 8 ms, thickness/gap = 2/1 mm) and coronal and sagittal T2-weighted TSE sequences (TR = 4000 ms, TE = 116 ms, thickness/gap = 2/1 mm).

### Imaging Processing

All the images were exported from the radiological department and transformed into the Neuroimaging Informatics Technology Initiative (NIFTI) format using Mango (v4.1) software, which is a standard format for image processing. The pituitary adenomas were delineated using 3D Slicer (v4.11.0), open-source software for medical image processing, on sagittal T1-weighted sequences with contrast and adjusted on coronal T1-weighted and T2-weighted sequences with contrast. Two experienced neurosurgeons independently contoured all tumors blindly with the semiautomated module in the software (14). A senior neurosurgical professor helped with the finalization of tumor segmentation if the discrepancy of tumor volume estimation was over 20% between the two neurosurgeons. The suprasellar and intrasellar parts of tumors were divided according to the diaphragm sella. If the sellar diaphragm was unclear due to the invasion of the tumor, the horizontal level of the “waist sign” was considered the boundary between the suprasellar part and the intrasellar part. The suprasellar volume (V1) and intrasellar volume (V2) were also calculated using 3D Slicer. The infrasellar parts of tumors, such parts invading into the sphenoid sinus and clivus were included in the intrasellar volume. The intercarotid distance was defined as the shortest distance between its inner surface on the layer in which the tumor had the longest horizontal diameter within the intrasellar part. Both the longest horizontal diameter of the tumor (D1) and the corresponding intercarotid distance (D2) were calculated using 3D Slicer. The V-D value was defined as the sum of two ratios, V1/V2 and D1/D2.

### Histological Analysis

Tumor samples were collected after transsphenoidal surgery, and routine H&E and immunohistochemical staining were performed in the Pathology Department in our hospital.

The Ki-67 value was reported as the percentage of tumor cells with positive nuclei in the lesion areas with the greatest labeling density, known as hotspots, using high-power fields (HPF 0.30 mm2, 400x magnification).

### Statistical Analysis

All data were analyzed using the R program (v4.0.3). The difference between the two groups was determined by Student’s t-test if the data had a normal distribution or a nonparametric test if the data did not have a normal distribution. The relationship between V-D value and clinical characteristics was assessed by Spearman’s correlation. The receiver operating curve (ROC) was constructed to estimate the predictive capacity of V-D system, and the area under curve (AUC) was also calculated using the “pROC” package in the R program. The Kaplan-Meier method was applied to assess the differences in the recurrence-free interval between patients grouped by the cutoff value of V-D system using “survival” and “survminer” packages in R. A p value of less than 0.05 indicated statistical significance.

## Results

### General Characteristics

There were 45 males and 53 females ranging from 14 to 79 years old included in this study. The clinical characteristics of the 98 NFPA patients are presented in **Table 1**. All patients signed informed consent forms, and the study met with approval from the ethics committee for Clinical Research and Animal Trials of The First Affiliated Hospital of Sun Yat-Sen University (Guangzhou, China). There were no significant differences between the nonrecurrent group (NR) and recurrent group (RE) in terms of age, sex, Hardy–Wilson grade, Ki-67 index, or other hormone levels, including PRL, testosterone, LH, and FSH. Knosp grade, tumor size, volumes, and diameters were significantly different between the two groups.

**Table 1:** Baseline information (data of normal distribution were shown as means (standard deviation), data that did not fit the normal distribution were shown as means (median, interquartile range).

	Non-recurrent	Recurrent	p
n	43	55	
Sex [Male(%)]	24 (55.8)	21 (38.2)	0.125
age [mean (SD)]	46.42 (14.53)	43.60 (12.59)	0.307
Knosp grade (%)			0.034
0	2 (4.7)	0 (0.0)	
1	16 (37.2)	9 (16.4)	
2	7 (16.3)	15 (27.3)	
3	12 (27.9)	18 (32.7)	
4	6 (13.9)	13 (23.6)	
Hardy grade (%)			0.279
I	13 (30.2)	7 (12.7)	
II	15 (34.9)	22 (40.0)	
III	8 (18.6)	17 (30.9)	
IV	6 (14.0)	8 (14.5)	
V	1 (2.3)	1 (1.8)	
Tumor size (%)			0.012
Giant (D1>3)	8 (18.6)	20 (36.4)	
Large (1.5<=D1 ≤ 3)	21 (48.8)	29 (52.7)	
Small (D1<1.5)	14 (32.6)	6 (10.9)	
Total tumor volume [median (IQR)]	2648.33 [873.97,6911.56]	7920.27 [5313.34, 6573.73]	<0.001
V2 (mm^3^) [median (IQR)]	1808.24 [873.97,5768.55]	4635.93 [3330.06, 8449.81]	0.002
V1 (mm^3^) [median (IQR)]	0.00 [0.00, 1184.99]	3085.51 [804.66, 7717.00]	<0.001
V1/V2 [median (IQR)]	0.00 [0.00, 0.28]	0.67 [0.22, 1.29]	<0.001
Diameter			
D1 (mm) [median (IQR)]	22.90 [18.05, 29.85]	30.30 [24.35, 35.80]	<0.001
D2 (mm) [median (IQR)]	24.30 [22.50, 28.55]	27.30 [24.70, 31.20]	0.043
D1/D2 [median (IQR)])	0.87 [0.76, 1.08]	1.05 [0.92, 1.27]	<0.001
V-D value [median (IQR)]	1.02 [0.76, 1.40]	1.88 [1.38, 2.41]	<0.001
Time of recurrence [median (IQR)]	–	14.00 [6.50, 32.50]	–
Ki-67 [median (IQR)]	1.00 [0.00, 1.00]	1.00 [1.00, 1.00]	0.142
PRL (ng/ml) [median (IQR)]	19.90 [13.38, 34.34]	20.52 [10.93, 38.98]	0.98
Testosterone (ng/ml) [median (IQR)]	0.58 [0.24, 1.45]	0.36 [0.21, 0.71]	0.1
LH (ng/ml) [median (IQR)]	1.94 [1.22, 3.40]	1.48 [0.70, 3.04]	0.097
FSH (ng/ml) [median (IQR)]	4.13 [2.45, 5.92]	4.23 [2.47, 6.36]	0.728

### The Relation Between the VD System and Clinicopathological Features

Neither the level of hormones nor the Ki-67 index was correlated with the V-D value (**Table 2**). However, there was a significant correlation between the V-D value and recurrence time. Univariate logistic regression showed that the suprasellar volume, total tumor volume, supra-intrasellar ratio, Knosp grade, maximum tumor diameter, tumor-ICA ratio, and V-D value had a significant correlation with the recurrence of NFPAs, while the levels of Ki-67 and other hormones had no statistical correlation with recurrence (**Table 3**). Furthermore, multivariate logistic regression was performed, and the results show that the supra-intrasellar ratio, tumor-ICA ratio, and V-D value significantly correlated with recurrence (**Table 4**).

**Table 2:** Correlation between V-D value, Ki-67 and other hormone levels.

Characteristic	V-D value	Ki-67	PRL (ng/ml)	Testosterone (ng/ml)	LH (ng/ml)	FSH (ng/ml)
V-D value	1	0.16751	0.152164	0.090707	-0.07162	0.211216
Ki-67	0.16751	1	-0.26028	0.068129	-0.1286	-0.04428
PRL (ng/ml)	0.152164	-0.26028	1	-0.01206	0.003982	0.164758
Testosterone (ng/ml)	0.090707	0.068129	-0.01206	1	0.071892	0.054833
LH (ng/ml)	-0.07162	-0.1286	0.003982	0.071892	1	0.612763
FSH (ng/ml)	0.211216	-0.04428	0.164758	0.054833	0.612763	1

**Table 3:** Univariate Logistic regression between recurrence and multiple factors.

	Coef	S.E.	Wald	P
V2 (mm^3^)	0.0002	0.0001	1.10	0.2692
V1 (mm^3^)	0.0005	0.0001	3.70	0.0002
Total tumor volume (mm^3^)	0.0001	0.0000	3.23	0.0012
V1/V2	2.6700	0.6537	4.08	<0.0001
Knosp grade	0.4133	0.1879	2.20	0.0278
Hardy grade	0.9556	0.9673	0.99	0.3232
Tumor size	2.5614	1.4856	1.72	0.0847
D1 (mm)	0.0973	0.0281	3.46	0.0005
D2 (mm)	0.0714	0.0413	1.73	0.0842
D1/D2	2.6388	0.8612	3.06	0.0022
Ki-67	0.4325	0.2709	1.60	0.1103
PRL (ng/ml)	0.0040	0.0090	0.44	0.6574
LH (ng/ml)	-0.0550	0.0677	-0.81	0.4160
FSH (ng/ml)	0.0039	0.0255	0.15	0.8769
Testosterone (ng/ml)	-0.1255	0.1154	-1.09	0.2770
V-D value	2.5738	0.5487	4.69	<0.0001

**Table 4:** Multivariate Logistic regression between recurrence and multiple factors.

	Coef	S.E.	Wald	P
V1 (mm^3^)	0.0002	0.0002	0.78	0.4381
Total tumor volume (mm^3^)	0.0000	0.0001	-0.35	0.7238
Knosp grade	-0.1657	0.2857	-0.58	0.5619
V1/V2	2.1663	0.9237	2.35	0.0190
D1	0.0788	0.0629	1.25	0.2101
D2	1.8152	0.8713	2.08	0.0372
V-D value	2.1021	0.6700	3.14	0.0017

### The Predictive Value of the VD System Recurrence Compared to the Hardy and Knosp Systems

**Figure 1:**
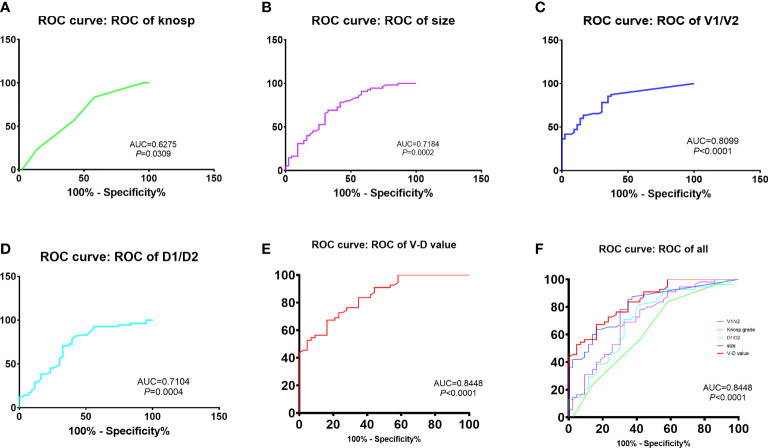
Comparison of recurrent time divided by the cut-off value of V-D: The predictive efficacy of knosp grades **(A)**, tumor size **(B)**, ratio of V1 and V2 **(C)**, ratio of D1 and D2 **(D)** and V-D value **(E)**. The V-D value has shown the best efficacy in predicting recurrence in NFPAs (AUC = 84.48%, sensitivity = 0.837, specificity = 0.673,cut-off value = 1.53).

The predictive effectiveness of the V-D value was evaluated using ROC analysis (**Figure 1**). The area under the curve (AUC) reached 84.5%, with a specificity of 67.3% and sensitivity of 83.7%, showing that the predictive value was high compared to that of the Knosp and Hardy–Wilson grades. The cutoff limit of the V-D value was 1.53.

### The Relation Between the VD System and Recurrence Time

**Figure 2:**
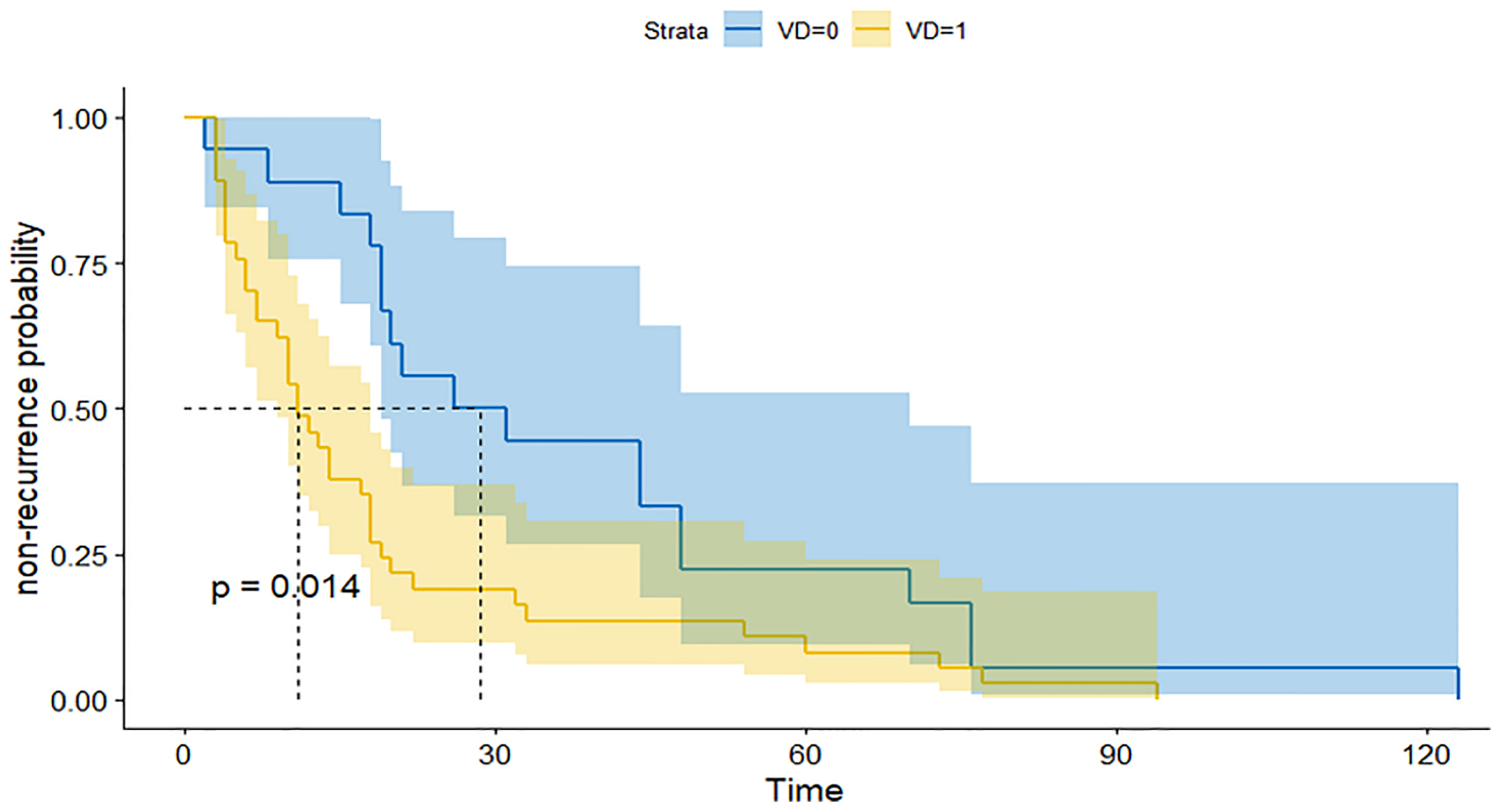
ROC for the prediction of recurrence by several indexes: The recurrent time of patients with high V-D value (yellow line) was significantly shorter than patients with low V-D value (blue line).

**Figure 3:**
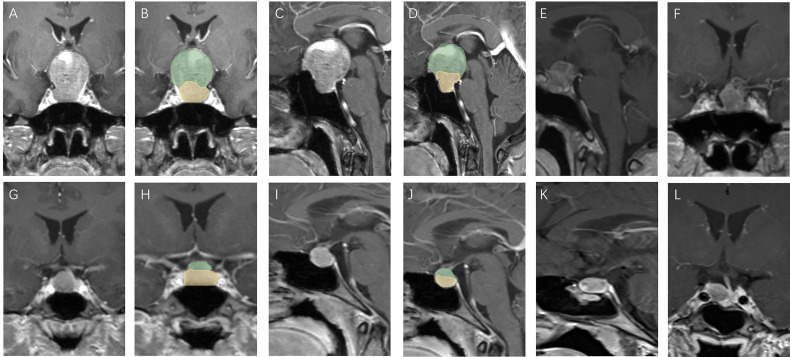
Patients with high V-D value: A 51 year old female, TV: 13699.48 mm^3^, V2: 1620.19 mm^3^, V1:12079.29 mm^3^, V-D: 8.64 **(A–D)**, relapse 13 months after surgery **(E, F)**; a 30 year old female, TV: 470.46 mm^3^, V2: 186.48 mm^3^, V1:283.98 mm^3^, V-D: 2.56 **(G–J)**, relapse 94 months after surgery **(K, L)**.

**Figure 4:**
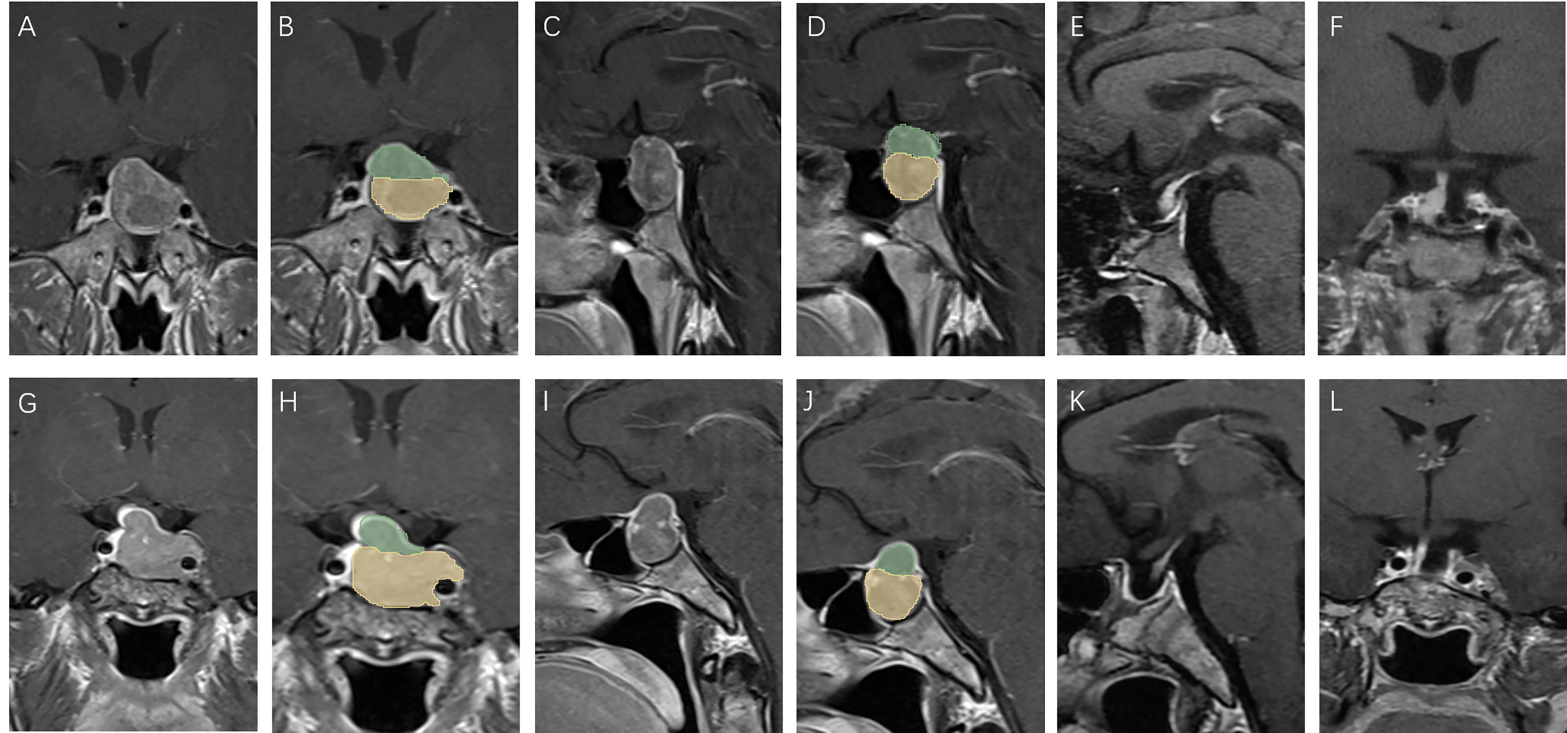
Patients with low V-D value: A 39 year old male, TV: 5781.19 mm^3^, V2: 4376.40 mm^3^, V1:1404.79 mm^3^, V-D: 1.19 **(A–D)**, no relapse in 7 years of follow-up **(E, F)**; a 43 year old female, TV: 5888.45 mm^3^, V2: 5188.17 mm^3^, V1:700.28 mm^3^, V-D: 1.31 **(G–J)**, no relapse in 7 years of follow-up **(K, L)**.

We further subdivided the recurrent group into a large group (V-D >1.53) and a small group (V-D <1.53) using the cutoff value of V-D. The survival curve of the two groups showed that the large group had a shorter recurrence time than the small group (**Figure 2**). Therefore, the V-D value can also reflect the probability of early recurrence in those patients (**Figures 3**, **4**).

## Discussion

NFPAs are benign intracranial tumors arising from adenohypophyseal cells. Patients usually visit the clinic much later than the time of disease onset, with symptoms caused by the compressed tissue surrounding the macroadenoma (1, 5). Therefore, the treatment and prognostic factors for NFPAs should be taken seriously. Our study has provided a brief and effective method for predicting the probability of recurrence in patients with NFPAs preoperatively, using some radiological indices.

Some studies have used molecular markers for the prediction of recurrence (15). However, pathology results can only be obtained after surgery, and few factors can predict recurrence before surgery. Predicting recurrence before surgery is essential not only for reducing patients’ distress but also for providing individual advice for their treatment. If a high potential of recurrence in a patient can be detected before surgery, a more aggressive surgery plan could be drawn up, and the follow-up plan adhered to more diligently. We found that a higher V-D value was positively related to tumor recurrence after surgery, showing good efficacy in the prediction of residual tumors (**Figures 3**, **4**). The V-D value can be obtained from preoperative radiological data and used in the preoperative discussion when seeking patient consent. For those with higher V-D values, surgeons can devise more radical strategies, such as resection of the internal wall of the cavernous sinus or extended transsphenoidal surgery. Intraoperative MRI detection can also be performed for those with higher V-D values to provide the greatest chance for achieving total resection.

In our study, patients with a V-D value higher than 1.53 were predicted to undergo relapse much earlier than those with a lower V-D value. On the one hand, this value represents a quantification of the difficulty of total resection during transsphenoidal surgery. Patients with residual tumors have a greater risk of progression postoperatively according to previous studies (5, 15–18). The relapse rate was higher after 10 years of follow-up than after only 5 years of follow-up in patients with a residual tumor. There was an interesting case in our study that showed the potential of quantifying surgical difficulty using the V-D value. A patient with a large pituitary adenoma (TV: 31626.76 mm^3^, V2: 1604.80 mm^3^, V1: 30021.96 mm^3^) only had a V-D value of 1.11 because the principal part of the tumor was located in the intra- and infrasellar regions, which were easily totally resected using endoscopes. Therefore, according to our results, follow-up should be performed for an indefinite period and more intensively for those with tumors with a high V-D value.

This value may reflect the growth pattern of tumors with higher invasiveness, which should be verified by further molecular studies. Ki-67 is a classical index for the description of tumor proliferation in pituitary adenomas (19, 20). However, current estimations of Ki-67 are mostly based on visual detection under a microscope, which lacks objectivity. In our study, the V-D value did not correlate with the level of Ki-67, suggesting that invasiveness or proliferation may not directly correlate with recurrence probability. As the examination of transcription factors was incomplete in our patients, we did not study the relationship. Further studies on molecular features such as the expression of pit-1, t-pit and p53, as well as the V-D value are necessary to provide deeper insights into the prediction methods.

With the development of artificial intelligence and data science, many studies have used radiomics methods or machine learning to predict the recurrence of brain tumors (18, 21, 22). However, models built by those methods lack universality between different study centers due to the heterogeneity of data collection, which is the key to the accuracy of model building. Although some models may show higher predictive value, they need complicated post-processing of radiological and clinical data. The V-D value provided in our study is rapidly calculated not only on some open-source software but also on many hospital platforms and even PACS systems in many centers. Different from the variability of radiomics features, the tumor volume and diameter will not change regardless of where the data are analyzed.

### Limitations

The first limitation of our study was the small sample size and lack of external validation. Larger and multicenter datasets should be analyzed further to verify our results in the prediction of recurrence in NFPAs. Second, as the patients in our center did not routinely undergo CTA or MRA before surgery, the carotid distance could only be roughly detected on common MRI for convenience. The carotid artery could be reconstructed using CTA or MRA preoperatively, to increase the accuracy of the D2 value in further studies. Third, most patients did not undergo detection of transcription factors, which could be used for further verification of the utility of the V-D value. Lastly, an automated algorithm for tumor segmentation will be needed for the accurate calculation of tumor volume in further studies.

## Conclusion

In conclusion, we discovered that there was a positive relationship between the V-D value and tumor recurrence of NFPAs. Patients with higher V-D values showed a higher probability of relapse and shorter recurrence time. Those with a higher V-D value should be given more attention in terms of surgery and follow-up.

## Data Availability Statement

The original contributions presented in the study are included in the article/supplementary material. Further inquiries can be directed to the corresponding authors.

## Ethics Statement

The studies involving human participants were reviewed and approved by Sun Yat-Sen University. The ethics committee waived the requirement of written informed consent for participation.

## Author Contributions

Conception and design: WC, MW, and CD. Drafting the article: WC and MW. Acquisition of data: WC and HW. Analysis and Interpretation of data: all authors. Critically revising the article: SY and HW. Approved the final version of manuscript on behalf of all authors: HW. Statistical analysis: WC and MW. Graphic visualization: WC and MW. Clinical diagnosis and radiological identification: WC, ZW, BH, and ZM. Administrative/technical/material support: WC, ZM, YZ, and HW. Study supervision: HW and YZ. All authors contributed to the article and approved the submitted version.

## Funding

The study was funded by Clinical Research Project of The East Division (the First Affiliated Hospital, Sun Yat-sen University, 2019004), Guangdong Natural Science Foundation (2018A0303130333), and The Chinese Postdoctoral Science Foundation (2019M663271).

## Conflict of Interest

The authors declare that the research was conducted in the absence of any commercial or financial relationships that could be construed as a potential conflict of interest.

## Publisher’s Note

All claims expressed in this article are solely those of the authors and do not necessarily represent those of their affiliated organizations, or those of the publisher, the editors and the reviewers. Any product that may be evaluated in this article, or claim that may be made by its manufacturer, is not guaranteed or endorsed by the publisher.
